# Alice M. Boring: a pioneer in the study of Chinese amphibians and reptiles

**DOI:** 10.1007/s13238-015-0165-1

**Published:** 2015-05-22

**Authors:** Shu Zheng

**Affiliations:** Institute for the History of Natural Science, Chinese Academy of Sciences, Beijing, 100190 China

Alice Middleton Boring (博爱理, 1883–1955) was an American biologist and herpetologist who spent much of her academic life in China. She devoted herself to improving biology research and education in China, and is a pioneer in the study of Chinese amphibians and reptiles (Figs. 1 and 2).

**Figure 1:**
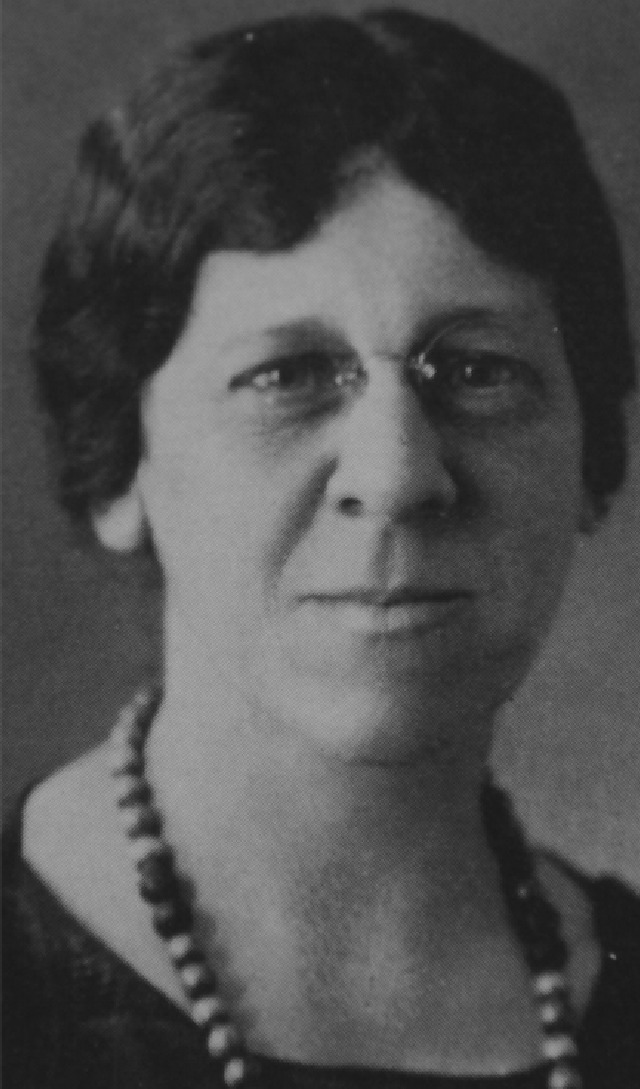
Alice M. Boring

**Figure 2:**
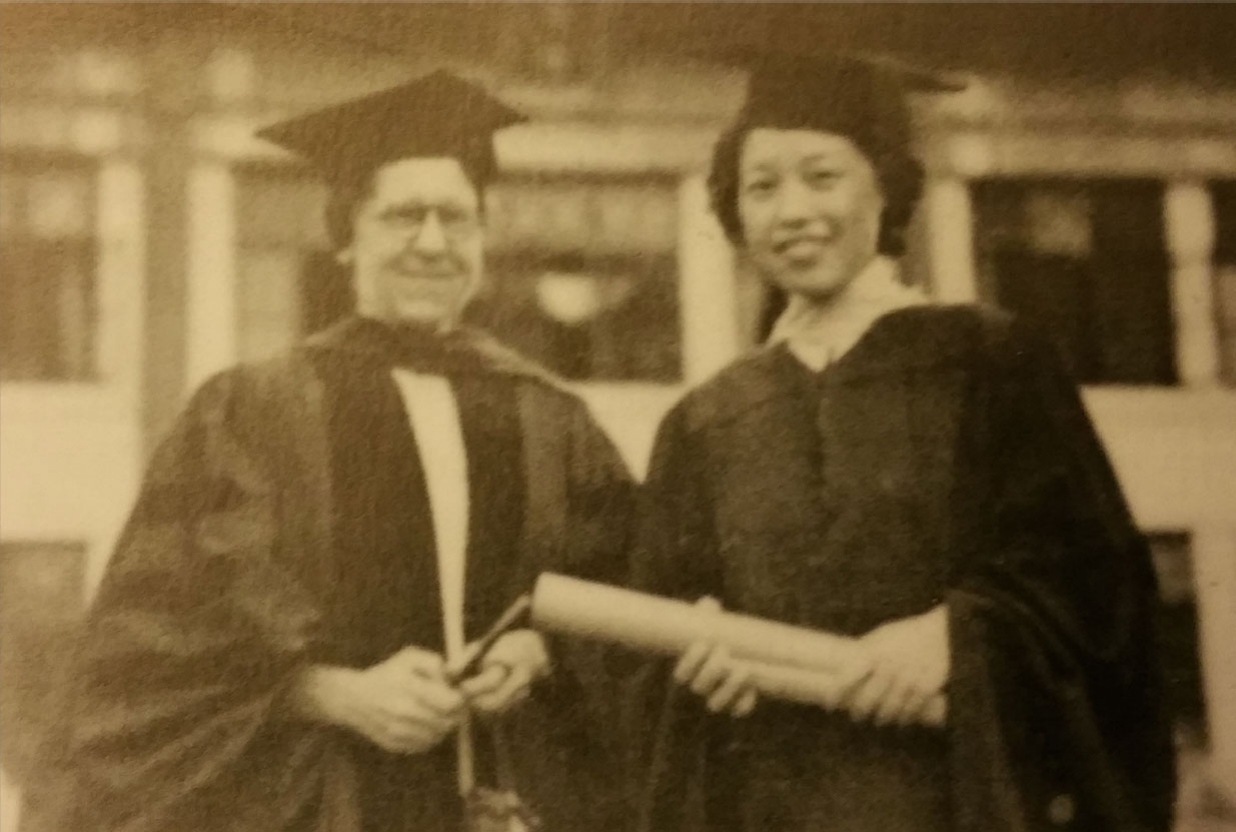
Alice M. Boring at Yenching University, 1939

**Figure 3:**
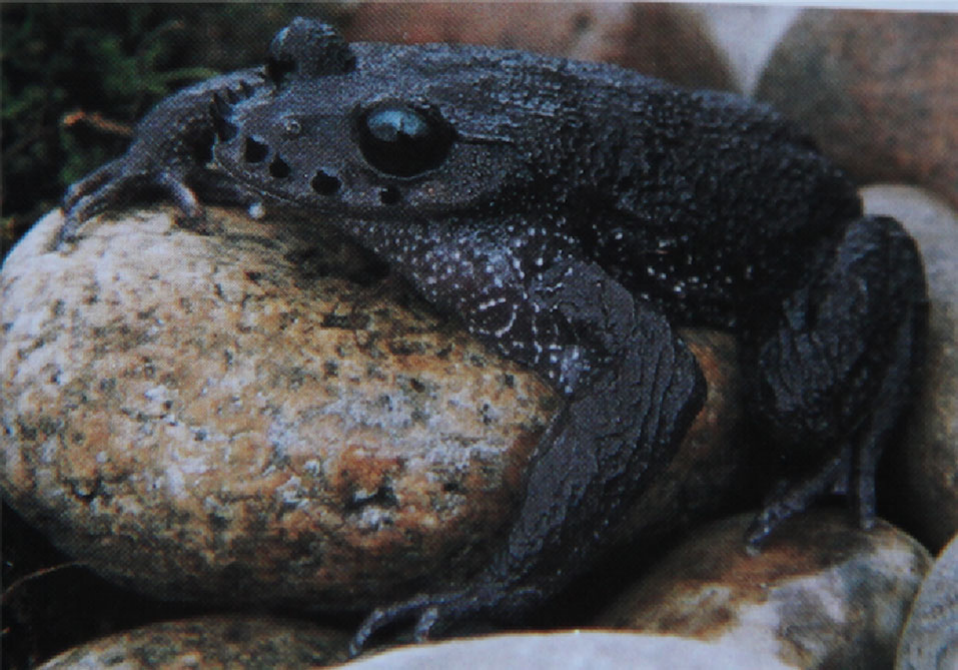
*Vibrissaphora boringii*

 Boring received her Ph.D. in 1910 from Bryn Mawr College, where she studied under biologists including Thomas Hunt Morgan and Nettie M. Stevens. After some years of teaching and researching biology in the United States, she started her career in China (Luo 2014). From 1918 to 1920, she worked as Assistant in Biology at Peking Union Medical College, which was funded by Rockefeller Foundation. In 1923, she was appointed the head of biology department at Yenching University, where she worked until Japanese closed the university in 1941. She returned to Yenching University in 1946, and stayed there till she went back to America in 1950.

Although originally trained and worked in the field of genetics and cytology, Boring’s academic interest shifted to taxonomy and distribution of reptiles and amphibians during her stay in China. The variety of Chinese herpetofauna offered her an exciting research field, where she worked and published with biologists Nathaniel Gist Gee, Clifford H. Pope and Hu jingfu (胡经甫), as well as her students. In order to collect specimens, Boring had expeditions in different parts of China, such as Jiangxi, Zhejiang, Anhui, etc. Boring’s hard work yielded substantial results. Her program in Yenching University became one of two major centers in China for the study of amphibians and reptiles (Adler and Zhao 1993). She was one of the charter members of Peking Natural History Society, and had twenty-one papers and one handbook published by the Society between 1929 and 1950 (Ogilvie et al. 1999, p. 110). In 1945, she published another important Bibliography: *Chinese Amphibians*: *Living and Fossil Forms*. Boring also provided American museums and scientists with specimens and data in China. Her efforts further broadened the global understanding of the Chinese amphibians and reptiles.

Apart from research, teaching was always Boring’s primary work in China. Her students described her as both strict and warm. She asked for very high standards when it came to laboratory operation and academic study. Behind her tough façade, she cared about her students warm-heartedly. She insisted to have dinner with every biology student at her house at least once a year. In order to prepare her students to study abroad, she would personally teach them western table manners (Ogilvie et al. 1999, p. 125). Boring’s students made outstanding contributions to medicine and biology in China and abroad. One of them is Liu Chengzhao (刘承钊, 1900–1976), who is considered to be a founder of Chinese herpetology. Liu once discovered a new species of toad in Sichuan Province in China, and named it *Vibrissaphora boringii*, to express his enormous gratitude to Boring (Yenching Institute 2001; Fig. 3)

It’s noteworthy that Boring’s life and career in China had always been challenged by the unstable social environment and inconvenient material condition. After giving up her secure life as an Associate Professor in an American university, she arrived in an oriental country full of civil strife. During the World War II, Boring insisted to continue her research and teaching in Yenching, in spite of the Japanese invasion. She had to stay in an internment camp before she was forced to leave China due to the war between America and Japan in 1943. She returned to China as soon as the chaos was over. There were reasons why she felt so “belong here” as she wrote to her family. Apart from her friendship with colleagues and students in China, her willing to fulfill herself by helping Chinese people with scientific knowledge transcended material hardship.

## References

[CR1] Adler K, Zhao EM (1993). Herpetology of China.

[CR2] Luo GH (2014) The development of modern biology in China. Beijing: China Science and Technology Press. pp. 78–80 (罗桂环. (2014) 中国近代生物学的发展. 北京:中国科学技术出版社. pp. 78–80)

[CR3] Ogilvie MB, Clifford J, Choquette A (1999). Dame full of vim and vigour: biography of alice middleton boring (1883–1955), an American Biologist in China.

[CR5] Yenching Institute (2001) Biographies of people in Yenching. Volume 1. Beijing:Beijing University Press. pp. 26–27 (燕京研究院编 (2001) 燕京大学人物志. 第一辑. 北京: 北京大学出版社. pp. 26–27)

